# The Contribution of Surrounding Margins in the Promotion of Natural Enemies in Mediterranean Apple Orchards

**DOI:** 10.3390/insects10050148

**Published:** 2019-05-23

**Authors:** Neus Rodríguez-Gasol, Jesús Avilla, Yahana Aparicio, Judit Arnó, Rosa Gabarra, Jordi Riudavets, Simó Alegre, Jaume Lordan, Georgina Alins

**Affiliations:** 1 IRTA Fruitcentre, PCiTAL, Park of Gardeny, Fruitcentre Building, 25003 Lleida, Spain; simo.alegre@irta.cat (S.A.); jaume.lordan@irta.cat (J.L.); georgina.alins@irta.cat (G.A.); 2 Department of Crop and Forest Science, Agrotecnio, University of Lleida, Avda. Alcalde Rovira Roure 191, 25199 Lleida, Spain; avilla@pvcf.udl.cat; 3 IRTA, Ctra de Cabrils km.2, 08348 Cabrils, Barcelona, Spain; yahanamichelle.aparicio@irta.cat (Y.A.); judit.arno@irta.cat (J.A.); rosa.gabarra@irta.cat (R.G.); jordi.riudavets@irta.cat (J.R.)

**Keywords:** agroecological infrastructures, biological control, flower strip, natural enemies, Syrphidae, parasitoids, *Eriosoma lanigerum*, *Dysaphis plantaginea*

## Abstract

(1) Habitat management can enhance beneficial arthropod populations and provide ecosystem services such as biological control. However, the implementation of ecological infrastructures inside orchards has a number of practical limitations. Therefore, planting/growing insectary plants in the margins of orchards should be considered as an alternative approach. (2) Here, we assessed the efficacy of a flower margin composed by four insectary plant species (*Achillea millefolium*, *Lobularia maritima*, *Moricandia arvensis* and *Sinapis alba*), which was placed on an edge of four Mediterranean apple orchards to attract natural enemies of two apple tree aphids (*Dysaphis plantaginea* and *Eriosoma lanigerum*). We also characterized the natural enemies present in the aphid colonies. (3) Our results show that the implementation of a flower margin at the edge of apple orchards attracts predators (Syrphidae, Thysanoptera, Araneae, Heteroptera, Coleoptera) and parasitoids. Parasitoids are the main natural enemies present in aphid colonies in our area. (4) The implementation of the flower margins successfully recruited natural enemy populations, and the presence of parasitoids in the surroundings of the orchards increased the parasitism of *D. plantaginea* colonies.

## 1. Introduction

Agriculture faces the challenge of responding to social demands for healthier food and more environmentally friendly practices while maintaining crop yields [1,2]. Consequently, agricultural practices that reduce reliance on conventional inputs and enhance ecosystem services, such as pollination or biological control, have received much research attention in recent years [3]. Habitat management has the potential to meet both agronomic and ecological objectives through the regulation of insect pest populations, often by intensifying the impact of the natural enemy community and through the preservation and promotion of biodiversity [4,5,6]. In this regard, fruit orchards are ideal agricultural landscapes in which to implement habitat management practices. In contrast to arable and vegetable crops, fruit trees remain in the orchard for several seasons, so there is a higher probability of conservation biological control succeeding [4,5,7]. Additionally, orchards are usually subjected to high pesticide use due to pest control [4]. Therefore, there is a need to explore other viable pest management options.

Aphids are major pests of apple (*Malus domestica* Borkh.) orchards under temperate climates, where they feed on the phloem of trees and cause an economic impact [8]. Several aphid species can infest apple trees, of which the two most damaging are the rosy apple aphid (RAA) *Dysaphis plantaginea* Passerini (Hemiptera: Aphididae) and the woolly apple aphid (WAA) *Eriosoma lanigerum* Hausmann (Hemiptera: Aphididae) [9]. RAA infestations affect the aerial parts of trees, causing leaf rolling, shoot twisting, chlorosis and deformation of fruits. Severe infestations can even affect the development of flower buds the following year and reduce overall tree vigor [8,10]. WAA infestations can occur both on the aerial and subterranean woody tissue of the trees, also causing deformations and galling on the roots, trunk, branches and twigs [11,12].

The presence of ecological infrastructures can favor the pollinator and natural enemy community and even improve the biological control of aphids, through the provision of shelter and food (pollen, nectar or alternative prey) [5,7,13]. These infrastructures should be positioned as close as possible to the orchards in order to achieve maximum conservation biological control [7]. However, the implementation of ecological infrastructures, such as flower strips, inside the orchards has major practical limitations due to the shredding of pruning waste, the mechanical control of groundcover, and water scarcity. After tree pruning, the cuttings, which are not removed and remain on the ground, are shredded in order to facilitate access to the orchards and the decomposition of the wood. In addition, the groundcover of orchards is usually mown/shredded to control the height of weeds and reduce competition for water and nutrients [14]. Therefore, auxiliary plants that are highly competitive or that grow too high are not suitable for orchard alleys [14,15]. With regard to water scarcity, the placement of flower resources in Mediterranean orchards presents an additional challenge, as the plants must be adapted to low water regimes, therefore limiting species selection. Hence, flower margins on the edges of orchards emerge as an alternative approach to alleys because they do not need to be mowed/shredded and facilitate irrigation management systems.

The implementation of flower strips in orchard margins has been shown to have positive effects on the population of aphid natural enemies [15,16,17,18]. However, to the best of our knowledge, no study has found an effect of the implemented flower strips in the abundance of natural enemies on aphid colonies. For this reason, a better understanding of the relationships between the natural enemies present in surroundings and the ones present in the aphid colonies is crucial to improve aphid biological control.

Thus, the objectives of our work were to: (1) assess the attractiveness of a specific flower margin to auxiliary fauna, (2) characterize the natural enemies of RAA and WAA present in Mediterranean apple orchards and (3) assess the influence of the surrounding margins in the RAA and WAA natural enemy populations present in the aphid colonies.

## 2. Materials and Methods

### 2.1. Study Area, Orchards and Treatments

The current study was conducted in 2015 and 2016 in the fruit tree-growing area of Lleida (Catalonia, NE Spain). This area is characterized by a semi-arid Mediterranean climate, with a mean annual rainfall of 350 mm.

Five organic apple orchards were surveyed for the study: orchards E1, E3, E4 and E5 in 2015 and orchards E1, E3, E5 and E6 in 2016 (Table S1). During the period of the study, the following pesticides were applied: azadirachtin (sprayed between the end of March and April to control RAA), granulosis virus (applied from May to August to control the codling moth (*Cydia pomonella* L. (Lepidoptera: Tortricidae)), and lime sulfur (applied from March to June to control apple scab (*Venturia inaequalis* Cooke)).

**Figure 1:**
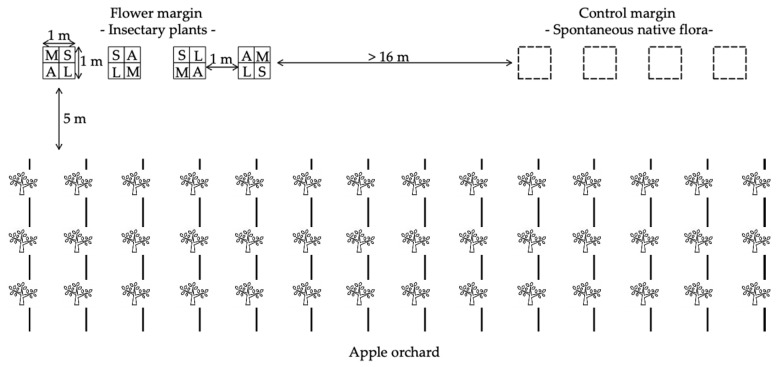
Setup of the experiment. A = *A. millefolium*, L = *L. maritima*, M = *M. arvensis*, S = *S. alba*.

In each orchard, two treatments were compared: a spontaneous margin and an implemented flower margin. The two margins were subdivided in four plots of 1 × 1 m, which were spaced 1 m from each other. Both margins were at least 16 m apart from each other and placed 5 m from the first tree of the row, perpendicularly to the rows (Figure 1).

The flower margin was formed by four insectary plant species: *Achillea millefolium* L. (Compositae), *Lobularia maritima* L. (Brassicaceae), *Moricandia arvensis* L. (Brassicaceae) and *Sinapis alba* L. (Brassicaceae) (Table S2). These plants were planted in plastic boxes of 50 cm length, 35.5 cm width and 31 cm height, and each box contained seven plants of one single species. Each plot had four boxes, one for each insectary species, which position was randomized per plot (Figure 1). The insectary plants were drip irrigated at the same frequency as the apple trees and the spontaneous weeds present in these boxes were removed fortnightly. The flower margins were established during the second half of March and were dismantled in October in both years.

The spontaneous margin was composed by native flora commonly found in the apple orchards of the area, which was mainly Gramineae (Table S3). In order to characterize the species present in both margins, fortnightly in 2015, the Braun-Blanquet cover-abundance scale [19] was used, and the phenology of each species was annotated.

### 2.2. Attractiveness of the Flower Margin to Natural Enemies and Phytophagous Insects

Visual samplings were performed to estimate the attractiveness of the margins to adult hoverflies. For three minutes per plot (12 minutes per margin), an observer counted the number of adult hoverflies hovering above or touching the flower of the insectary or spontaneous plant species present in a plot. The observations were conducted under favorable weather conditions: sunny to lightly overcast days, with no or low wind speed (0–4.2 m/s) and temperature above 15 °C.

Beating tray samplings (BTS) were conducted to estimate the attractiveness of the margins to natural enemies (except adult hoverflies) and phytophagous insects. BTS consisted of three consecutive beats with the hand. In each plot, four BTS were taken after visual observations had been made. In the flower margin, each BTS corresponded to an insectary plant species, while in the control margin the beatings were made on flowering species whenever possible. In the latter case, the plant species where the BTS was conducted were noted. The arthropods falling as a result of the BTS were collected on a white tray (24 × 35 cm) and classified into the following groups: predators (Araneae, predatory Thysanoptera, predatory Coleoptera, predatory Heteroptera, Syrphidae larvae, Chrysopidae, Cecidomyiidae, Trombidiidae and Forficulidae), parasitoids (both adults and mummies), and phytophagous insects (Aphididae, phytophagous Thysanoptera and phytophagous Heteroptera). Individuals were returned to the margin after visual identification to family or order in the case of spiders (Table S4).

Both visual samplings and BTS were conducted fortnightly from April (petal fall) to September in 2015 and 2016. In both years, the samplings were performed on coincident weeks.

### 2.3. Assessment of D. plantaginea, E. lanigerum and Their Natural Enemies

To assess the presence of RAA, WAA and their natural enemies, 400 shoots per orchard (10 shoots per tree in 40 randomly selected trees, placed up to 30 m away from the margins) were revised. The shoots that were infested by any of the two mentioned aphids and were examined for natural enemies, which were removed after being quantified and identified. The number of aphids was not quantified since the assessment of biological control was not among our objectives. Then, the shoots were cut, kept in plastic glasses with fabric lids and left at the laboratory (at 20 °C temperature and 12:12 h (L:D)) for two weeks to allow unseen immature stages of the insects to develop. Then, the natural enemies were quantified and identified with the same criteria as in the other samplings (Table S4). In addition, in the case of parasitoids found in the RAA colonies, a sample of 16 individuals was randomly selected and identified to genera and species level, when possible with taxonomic keys by Barahoei et al., [20] and Rakhshani et al., [21]. These samplings were conducted fortnightly in 2016.

### 2.4. Data Analysis

In order to evaluate the attractiveness of the margins to natural enemies and phytophagous insects, two analysis were performed: a weekly and a global analysis. For the weekly analysis, the average of the four plots per treatment and orchard was calculated. For the global analysis, the average of those 13 weeks per treatment and orchard was calculated. In both analysis, data from the visual observations and the BTS were referred as the number of arthropods per area and time (number/ (1 m^2^ × 3 min)), and the number of arthropods per area (number/1 m^2^), respectively. Response variables were modeled using linear mixed effect models where year and treatment were fixed factors and orchard was a random factor. Each orchard was considered as a replication (n = 8). Interaction between factors was considered. Residual analysis was performed to ensure that model assumptions were met and if necessary, data were log transformed.

In order to describe the natural enemies present in RAA and WAA colonies, the beneficial arthropods sampled from the aphid colonies in each week were referred to as the number of natural enemies per colony.

In order to assess the relationship between the arthropods found in the control and the implemented margin, correlations were run between these groups of arthropods: adult hoverflies recorded in the visual observations and predators, parasitoids and phytophagous insects captured in the BTS. In this case, all data from 2015 and 2016 were used (April to September). In contrast, for the correlations between the arthropods found in the margins and the ones present in the aphid colonies, only the dates when aphids were found in the orchard were used for the analysis. In the case of the RAA colonies, data from April to June of 2016 was used. Correlations were run between: the percentage of shoots infested with RAA, the number of Syrphidae predators, non-Syrphidae predators and parasitoids per infested shoot, the number of adult hoverflies assessed in the visual observations of the margins and the number of predators, parasitoids and phytophagous insects collected by the BTS of the margins. In the case of the WAA colonies, data from May to August of 2016 was used to run the correlations between the percentage of shoots infested by WAA, the number of predators and parasitoids per infested shoot, and the number of predators, parasitoids and phytophagous insects found in the flower and control margin. Data collected per week and orchard was analyzed by Spearman correlation coefficients. 

A significance level of *p* ≤ 0.05 was considered for all the analysis. Data were analyzed using the JMP statistical software package (Version 13; SAS Institute Inc., Cary, NC, USA).

## 3. Results

### 3.1. Attractiveness of the Margins to Natural Enemies and Phytophagous Insects

During the whole sampling period, a total of 2011 visits by hoverflies were counted in the visual observations, and 4772 predatory arthropods, 1024 parasitoid wasps and 11,778 phytophagous insects were found in the BTS.

Overall, the most abundant predators captured in the BTS were Heteroptera (46.7%), Araneae (34.2%), Thysanoptera (8.9%) and Coleoptera (6.3%). Among the predatory Heteroptera, Anthocoridae and Miridae were the most abundant and most of the predatory Coleoptera found belonged to Coccinellidae. Other groups like Trombidiidae (1.9%), Chrysopidae (1.0%), Syrphidae larvae (0.8%), Forficulidae (0.3%) and Cecidomyiidae (0.1%) were scarce and were grouped as “Other predators” for the statistical analysis. Parasitoids captured in the BTS could not be identified to lower taxonomic groups. The phytophagous insects found in the margins were mainly Aphididae (45.6%), phytophagous Tysanoptera (41.9%), and Pentatomidae (12.3%). The presence of phytophagous Colepotera was sporadic (0.2%).

Data of the average number of arthropods per week and orchard recorded from the different sampling methods were pooled for the whole sampling period to assess the attractiveness of the flower margin to the diverse arthropod groups. Overall, the mean number of adult hoverflies was significantly higher in the flower margin than in the control margin (Table 1). Neither significant interactions nor significant differences between years were found in the case of the adult hoverflies (Table 1). In the case of the BTS, significant quantitative interactions between year and treatment were only found in the case of predatory Coleoptera. Also, significant differences between years were found in the case of predatory Thysanoptera and predatory Coleoptera, which were more abundant in 2015 and 2016, respectively (data not shown). The mean number of the groups of natural enemies (“All predators”, “Thysanoptera”, “Araneae”, “Heteroptera”, “Coleoptera” and “Parasitoid wasps”) was significantly higher in the flower margin in all the cases except from the group “Other predators” (Table 1). Neither significant interactions nor significant differences between years were found for the phytophagous insects captured in the margin, and these were more abundant in the flower margin than in the spontaneous one (Table 1).

**Figure 2:**
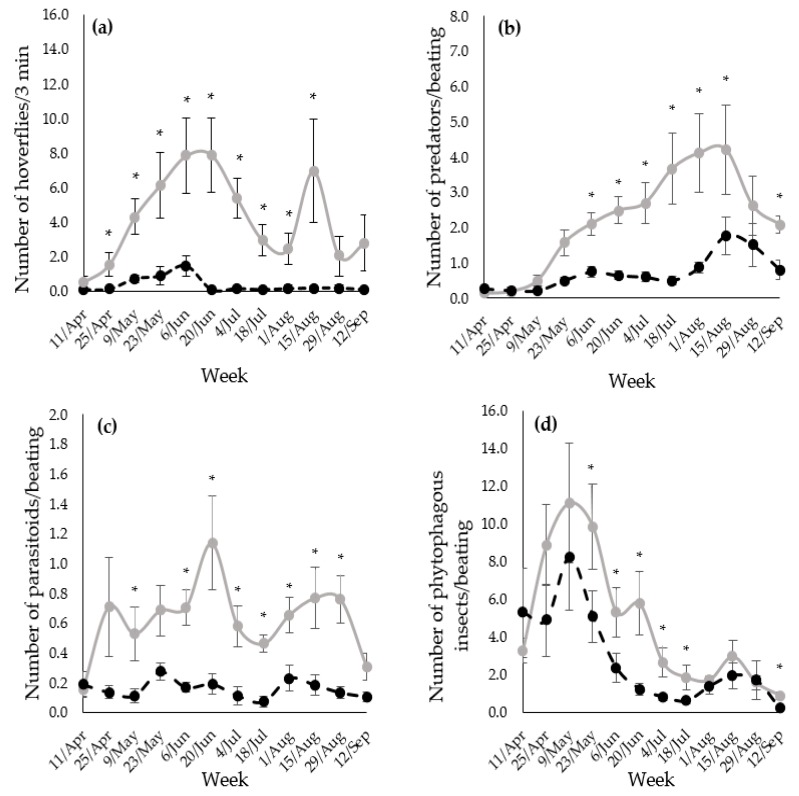
(**a**) Number of adult hoverflies per three minutes, (**b**) number of predators per beating, (**c**) number of parasitoid wasps per beating, and (**d**) number of phytophagous insects per beating in the flower margin (grey line) and in the spontaneous margin (black dashed line). Vertical bars show standard error. Data presented as average of 2015 and 2016 per week in 1 m^2^. ns = *p* >0.05, *= *p* <0.05.

In addition, data were analyzed by week in order to discern temporality in the attractiveness of the margins to arthropods. In the case of the adult hoverflies recorded during the visual observations, these were significantly higher in the flower margin than in the control margin most of the weeks (Figure 2a). In the case of the BTS, the number of predators, parasitoids and phytophagous insects was also significantly higher in the flower margin for most of the weeks. Predators were significantly higher in the flower margin from June to September (Figure 2b). In contrast, parasitoid wasps (Figure 2c) and phytophagous insects (Figure 2d) were more abundant in the flower margin during the middle of the sampling season. Statistically significant interactions between treatment and year, and significant differences between years were occasional (Table S5).

### 3.2. Assessment of D. plantaginea, E. lanigerum and Their Natural Enemies

The most abundant natural enemies present in RAA and WAA colonies were parasitoids. However, the diversity and relative abundance of the different groups of natural enemies differed between aphid species.

**Figure 3:**
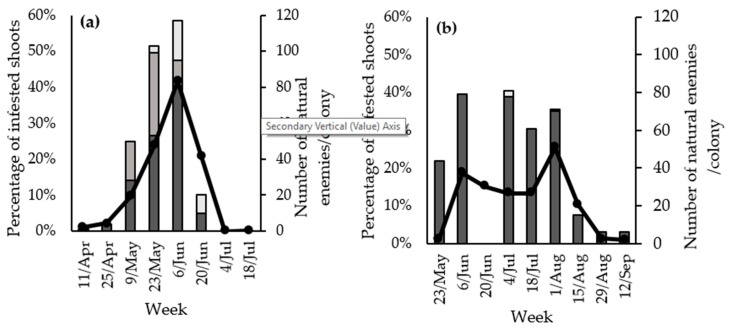
(**a**) Population dynamics of the RAA (black line) and their natural enemies per colony found in the colonies per week in 2016. Parasitoids (dark grey), Syrphidae (medium grey) and other predators (Miridae, Forficulidae, Araneae, Coccinellidae, Cecidomyiidae and Chrysopidae pooled together) (soft grey). (**b**) Population dynamics of the WAA (black line) and their natural enemies per colony found in the colonies per week in 2016: *A. mali* (dark grey) and other predators (Miridae and Coccinellidae pooled together) (soft grey). Primary axis shows percentage of shoots infested with RAA or WAA colonies. Secondary axis shows number of natural enemies per colony.

In the case of RAA, 173 parasitoids and 119 predators were found during the entire sampling period. Ichneumonoidea accounted for the whole diversity of the parasitoids present in RAA colonies. These were the only group of natural enemies found in April. Moreover, from early May to the end of June, Ichneumonoidea accounted for about 30–50% of the assemblage of natural enemies (Figure 3a). With regard to the species, 94% of the parasitoids were *Aphidius* spp. and the rest were *Ephedrus persicae* Froggatt (Hymenoptera: Braconidae). When *Aphidius* specimens could be identified to the species level, they were found to belong to *Aphidius matricariae* Haliday (Hymenoptera: Braconidae).

Hoverflies (Syrphidae) were the most abundant predators in RAA colonies (69.75%), followed by Cecidomyiidae (19.97%), Coccinellidae (6.72%) and Miridae (4.20%). Chrysopidae (1.68%), Araneae (0.84%) and Forficulidae (0.84%) abundance was minimal. Syrphidae were the first predators to reach the colonies, being the most abundant during May, while the rest of the predators appeared in late May and increased in abundance from then onwards (Figure 3a). The abundance of the natural enemies associated with the RAA colonies presented a similar pattern to that of RAA in the orchards: both increased from April to the beginning of June and decreased from then onwards (Figure 3a).

In the case of WAA, 359 parasitoids (all *Aphelinus mali* Haldeman (Hymenoptera: Aphelinidae) and four predators (one Miridae, two Coccinellidae and one Chrysopidae) were collected from the colonies (Figure 3b). The population dynamics of WAA natural enemies was similar to that of WAA: they appeared at the end of May, were abundant during June and July, and decreased thereafter (Figure 3b).

### 3.3. Correlations between the Natural Enemies and D. plantaginea and E. lanigerum Colonies

#### 3.3.1. Arthropods Found in the Margins

Significant positive correlations were found between the number of Syrphidae (hoverfly adults from the visual observations) and the other natural enemies recorded in the flower margin. Moreover, the number of adult hoverflies found in the flower margin was positively correlated with the number of phytophagous insects found in the flower margin and in the control margin. In contrast, no significant correlations were found for the number of adult hoverflies from the control margin and the number of phytophagous (Table 2).

The number of predators and parasitoids from the flower margin was positively correlated with the number of all the natural enemy groups from the same margin, and also with their respective groups of the control margin. Moreover, significant negative correlations were found between the number of predators found in the control margin and the number of phytophagous insects from the flower margin. The number of parasitoids captured in the flower margin was also positively correlated with the number of phytophagous insects found in the same margin, and the same occurred for the parasitoids form the spontaneous vegetation (Table 2). 

#### 3.3.2. Arthropods Found in the D. plantaginea Colonies

In the case of the RAA colonies, two groups of predators were considered according to their relative abundance in the aphid colonies: Syrphidae predators and non-Syrphidae predators. Correlations were run between the percentage of infested shoots and the natural enemies found in the RAA colonies (Syrphidae predators, non-Syrphidae predators and parasitoids).

During the period when RAA colonies were present in the orchard, no biologically significant correlations were found between hoverflies in the RAA colonies and the rest of arthropods from the margins (Table 3). On the other hand, significant positive correlations were found between the hoverflies of the RAA colonies and: 1) the percentage of shoots infested with RAA and 2) the parasitoids from the RAA colonies (Table 3). When correlations were run for non-Syrphidae predators from the RAA colonies, significant positive correlations were found between these and the phytophagous insects from the control margin (Table 3). In the case of the parasitoids found in the RAA colonies, significant positive correlations were found with: 1) adult hoverflies and predators from the flower margin, 2) the predators and parasitoids form the control margin and 3) the percentage of shoots infested with RAA (Table 3).

#### 3.3.3. Arthropods Found in the *E. lanigerum* Colonies

During the period when the WAA colonies were present in the orchard, no significant correlations were found between the predators found in the WAA colonies and those found in the margins (Table 4). Nonetheless, significant positive correlations were found between the parasitoids from the WAA colonies and the percentage shoots infested with WAA (Table 4).

## 4. Discussion

Adult hoverflies, predators and parasitoids were highly attracted to the flower margin implemented adjacent to the apple orchards when compared to a spontaneous margin. The floral composition of the margins may explain these differences: the spontaneous margin was mainly composed by Gramineae whereas the flower margin had more abundance of floral resources. In agricultural landscapes, orchard groundcover provides few or no floral resources due to the practice of mowing [22,23]. Hence, it is highly probable that the implemented flower margin represented a source of food (pollen, nectar or alternative prey) or shelter [5,7]. Thus, coinciding with previous studies [16,17,24,25,26,27], the presence of the flowering insectary plants in orchard margin has boosted resource availability, which may explain the attraction of natural enemies present in the area like parasitoids, spiders, Chrysopidae, Coccinellidae and Syrphidae ultimately benefiting their populations. Even though the addition of insectary plants can provide multiple benefits, a rigorous selection of the plant species is crucial in order to avoid the ones that harbor crop pests or diseases [5]. Furthermore, the implemented flower margin reflected most of the natural enemies and phytophagous present in spontaneous vegetation of the edge of the orchard since they were positively correlated with the beneficials and phytophagous of the control margin. However, in the case of adult hoverflies this effect was not observed. In our opinion, this highlights two biological traits of these flies: high dependence on flower resources (they require pollen and nectar in order to be able to reproduce and survive) [28,29] and high flying ability [30]. In the case of parasitoids, our data suggest that the phytophagous present in the margins could increase the host availability for parasitoids, since positive correlations were found between these groups.

With regard to the aphid colonies, RAA populations appeared in the orchards in early April and increased their presence until the beginning of June, when they migrated to their summer host (*Plantago* spp. Plantaginaceae). The populations of natural enemies associated with these colonies showed a similar trend, although the relative importance of the distinct assemblages changed with time. Despite the natural enemy diversity that we found in the RAA colonies is consistent with the findings of other studies [31,32,33], their relative abundance is different and it could be affected by the type of climate. Parasitism is likely to be favored by higher temperatures [34]. In fact, in studies carried out in Mediterranean areas [18,32,33] parasitoids represent an important part of the natural enemy guild, accounting for about the 30–50% of the beneficials present in the RAA colonies. While in colder climates, predators become more important relegating the importance of parasitoids [35,36,37]. In our study, Ichneumonoidea parasitoids were the first beneficial arthropods to colonize the aphid colonies, appearing one month earlier than predators and maintaining their presence for the whole infestation season. When identification to species level was possible, parasitoids mainly belonged to *A. matricariae* and *E. persicae* species. Both species have been cited as parasitoids of RAA in Europe, although little is known about their efficiency in suppressing populations of these aphids [33,38,39,40,41]. Due to our sampling method, we were not able to identify to species the parasitoids collected from the margins. Hence, we cannot confirm that the species that we collected from the RAA colonies were also present in any of our margins. However, the spontaneous vegetation seems to have a stronger influence than the implemented margin in the parasitoids present in the RAA colonies; only significant correlations were found between the spontaneous vegetation and the parasitoids present in the RAA colonies. In fact, we expected a positive correlation between the parasitoids collected in the flower margin and the ones present in RAA colonies since, in lab conditions, it was demonstrated that *L. maritima* increases the longevity of other species from the genera, such as *Aphidius ervi* Haliday (Hymenoptera: Braconidae) [42]. These results highlight the importance of field trials to determine the real contribution of the parasitoids present in the area on aphid control and how plant species can boost their presence.

Concerning the predators found in the RAA colonies, hoverflies were the most abundant group, accounting for more than half of the predators found in the colonies. In addition, they were the first predators present in the RAA colonies and they showed large populations during May. From mid-June onwards hoverflies stopped colonizing the RAA colonies, which can be attributed to the flies not perceiving the colonies as an adequate food source for their offspring. Adult hoverflies are known to show preferences in regard to aphid species and oviposition sites [43]. Therefore the lack of colonization from mid-June onwards could be explained by the presence of winged forms in the aphid colonies, which may signal an inappropriate food source for hoverfly offspring, as larvae show little mobility and may not be able to find an alternative feeding site if the colony disappears [43]. 

Despite our implemented flower margin being highly attractive to adult hoverflies, we failed to identify any correlations between the hoverflies present in the margins and the ones present in the RAA colonies. Nonetheless, the Syrphidae found in the RAA colonies, as well as the parasitoids, were positively correlated with the percentage of shoots infested with RAA. Previous studies have reported the attraction of Syrphidae and other natural enemies to herbivore-induced plant volatiles, e.g., Methyl salicylate [44], and aphid alarm pheromone components, e.g., E-(β)-farnesene [45]. Hence, it seems that the natural enemies present in the area were attracted to chemicals released by the RAA colonies. On the other hand, positive correlations were found between Syrphidae and parasitoids in the same colonies, which suggests that their populations were able to keep growing despite intraguild predation might be occurring between them [46,47]. 

Non-Syrphidae predators (Cecidomyiidae, Coccinellidae, Miridae, Chrysopidae, Forficulidae and Araneae) appeared about two weeks later than hoverflies and gradually increased in presence until the RAA colonies migrated. In our case, hoverflies were the main predator and the first to reach the RAA colonies, in agreement with Dib et al., [33] and Miñarro et al., [31]. As such, they represent a key group during the key period of RAA control. In contrast, the rest of the predatory assemblage appeared later, when RAA abundance was almost peaking, so their capacity to prevent RAA outbreaks is unlikely. However, since RAA has two hosts [10], the action of natural enemies during this period should not be underestimated, as they may contribute to reduce the abundance of summer migrants to the secondary host. As a consequence, the populations of the autumn migrants that recolonize apple trees might also be diminished. 

With regard to WAA populations, these were present in the orchard from late May to September. In this case, *A. mali* was the main beneficial arthropod found in the colonies, while the presence of predators was anecdotal. Even though earwigs, which are one of the most important WAA predators [15,48,49], are present in our study area [50], we failed to find them because our sampling method was not appropriated to assess this predator. Earwigs feed at night and remain concealed during the day [51], so night visual samplings would be required to detect the predatory activity of this insect on WAA colonies. In the case of hoverflies, we did not find any individual predating on WAA. Although some species of hoverflies feed on this aphid, they have never been reported as present in our area [52,53,54]. About parasitoids, *A. mali* is known to provide effective biocontrol of WAA on the aerial parts of the host plant [55,56] and moreover, its action can be consistently improved when other predators are also present [57,58,59]. However, in our case no correlation was found between *A.mali* and predators from the WAA colonies because, as mentioned above, our sampling method was not suitable to assess predators. On the other hand, our data suggests that neither the spontaneous vegetation nor the flower margin were used by *A. mali* because there was no correlation between the parasitoids present in the margins and the ones collected from WAA colonies. To the best of our knowledge, *A. mali* has not been previously reported to use floral resources and it is possible that adults are able to feed via host feeding [60,61]. Basic research is needed to know the feeding requirements of *A. mali* adults.

## 5. Conclusions

In conclusion, our study highlights the capacity of the implemented flower margin to gather natural enemy populations in the edge of apple orchards due to an enhanced alimentary and shelter supply. Parasitoids and hoverflies were the most important natural enemies present in the RAA colonies. In addition, the more parasitoids were present in the spontaneous vegetation, the more parasitoids were found in the RAA colonies. In contrast, no relationships was found between the natural enemies present in the flower margins and the ones present in the aphid colonies, probably because of the size of the flower margin. These results emphasize the importance of promoting these beneficials in the surroundings of the orchards. In contrast, *A. mali*, which is a specific parasitoid of WAA colonies and the most important natural enemy we found, did not benefit by the presence of additional resources. Hence, more research is needed to figure out how to enhance the abundance of this parasitoid. Further attention should be devoted to overcoming the technical difficulties associated with the implementation of flower strips (such as placement, irrigation, seeds availability and scale costs) and to unravelling the contribution of these zones to aphid control and ecosystem services.

## Figures and Tables

**Table 1:** Number of natural enemies (± Standard Error) assessed during all the sampling period in the visuals observations (VO, number/1 m^2^ × 3 min) and the beating tray samplings (BTS, number/1 m^2^) of the margins.

Type of Sampling	Arthropod Group	Treatment				Year		Year*Treatment	
		Control Margin ± SE	Flower Margin ± SE	F1,12	*p*	*F*_1,12_	*p*	*F*_1,12_	*p*
VO	Adult hoverflies	0.384 ± 0.077	4.242 ± 0.463	109.052	<0.001	0.968	0.348	3.182	0.114
BTS	All predators	0.724 ± 0.122	2.203 ± 0.332	39.991	**<0.001**	1.323	0.278	0.773	0.405
	Thysanoptera	0.035 ± 0.012	0.224 ± 0.039	42.784	**<0.001**	6.663	**0.026**	2.811	0.128
	Araneae	0.321 ± 0.049	0.679 ± 0.140	6.340	**0.034**	0.175	0.684	0.314	0.589
	Heteroptera	0.280 ± 0.114	1.085 ± 0.307	17.482	**<0.001**	4.676	0.057	0.230	0.644
	Coleoptera	0.045 ± 0.009	0.139 ± 0.048	6.100	**0.038**	7.036	**0.023**	5.506	**0.046**
	Other predators	0.044 ± 0.015	0.074 ± 0.007	3.810	0.081	2.003	0.185	3.446	0.094
	Parasitoid wasps	0.161 ± 0.015	0.623 ± 0.083	30.017	**<0.001**	0.282	0.606	0.799	0.394
	Phytophagous insects	2.828 ± 0.604	4.66 ± 0.598	7.655	**0.025**	1.654	0.229	0.002	0.968

* Test statistics (*F*-value (*F*)) and *p* (likelihood ratio) are shown. Significant *p* values (*p* <0.05) are shown in bold.

**Table 2:** Spearman correlation coefficients between: adult hoverflies (Syrphidae) from the visual observations (VO), predators, parasitoids and phytophagous insects from the beating tray samplings (BTS)) found in the flower margin (FM) and in the control margin (CM).

Type of Sampling		Arthropod Group	Syrphidae in the FM		Syrphidae in the CM		Predators in the FM		Predators in the CM		Parasitoids in the FM		Parasitoids in the CM	
			ρ	*p*	ρ	*p*	ρ	*p*	ρ	*p*	ρ	*p*	ρ	*p*
**FM**	**VO**	**Syrphidae**	−	−	0.192	0.06	0.344	**<0.001**	−0.025	0.806	0.38	**<0.001**	0.01	0.92
	**BTS**	**Predators**	0.344	**<0.001**	0.038	0.716	−	−	0.446	**<0.001**	0.514	**<0.001**	0.08	0.937
		**Parasitoids**	0.38	**<0.001**	0.185	0.072	0.514	**<0.001**	0.214	**0.036**	−	−	0.32	**0.002**
		**Phytophagous insects**	0.434	**<0.001**	0.183	0.075	−0.124	0.229	−0.383	**<0.001**	0.273	**0.007**	0.122	0.236
**CM**	**VO**	**Syrphidae**	0.192	0.06	−	−	0.038	0.716	0.027	0.793	0.184	0.072	0.126	0.221
	**BTS**	**Predators**	−0.025	0.806	0.027	0.793	0.446	**<0.001**	−	−	0.214	0.036	0.176	0.09
		**Parasitoids**	0.01	0.92	0.126	0.221	0.008	0.937	0.176	0.086	0.32	**0.002**	−	−
		**Phytophagous insects**	0.302	**0.003**	0.158	0.125	−0.125	0.227	−0.193	0.06	0.129	0.212	0.234	**0.022**

* Test statistics (Spearman correlation coefficients (ρ) and likelihood ratio (p)) are shown. Significant *p* values (*p* <0.05) are shown in bold.

**Table 3:** Spearman correlation coefficients (ρ) between the percentage of infested shoots and the number of natural enemies (Syrphidae predators, non-Syrphidae predators and parasitoids) found in the RAA colonies and the number of arthropods found in visual observations (VO) and beating tray samplings (BTS) in the flower margin (FM) and control margin (CM).

Type of Sampling		Arthropod Group	Syrphidae Predators in the RAA Colonies		Non-Syrphidae Predators in the RAA Colonies		Parasiotids in the RAA Colonies	
			ρ	*p*	ρ	*p*	ρ	*p*
FM	VO	Syrphidae	0.312	0.138	0.333	0.104	0.621	**0.001**
	BTS	Predators	0.309	0.142	0.146	0.498	0.488	**0.016**
		Parasitoids	0.173	0.418	0.272	0.188	0.281	0.183
		Phytophagous insects	0.312	0.138	0.025	0.908	0.226	0.289
CM	VO	Syrphidae	0.253	0.234	−0.039	0.854	0.105	0.626
	BTS	Predators	0.175	0.414	0.155	0.471	0.428	**0.037**
		Parasitoids	0.449	**0.028**	0.244	0.250	0.483	**0.017**
		Phytophagous insects	0.116	0.589	0.414	**0.040**	0.031	0.887
RAA	RAA colonies	% shoots infested with RAA colonies	0.536	**0.007**	0.233	0.273	0.606	**0.002**
		Syrphidae predators	−	−	−0.057	0.793	0.658	**<0.001**
		Non-Syrphidae predators	−0.057	0.793	−	−	0.191	0.373
		Parasitoids	0.658	**<0.001**	0.191	0.373	−	−

* Test statistics (Spearman correlation coefficients (ρ) and likelihood ratio (p)) are shown. Significant p values (*p* <0.05) are shown in bold.

**Table 4:** Spearman correlation coefficients (ρ) between the number of predators and parasitoids found in the WAA colonies and the number of arthropods found in visual observations (VO) and beating tray samplings (BTS) in the flower margin (FM) and control margin (CM).

Type of Sampling		Arthropod Group	Predators in the WAA Colonies		Parasitoids in the WAA Colonies	
			ρ	*p*	ρ	*p*
FM	VO	Syrphidae	0.298	0.078	0.181	0.292
	BST	Predators	0.139	0.419	0.055	0.752
		Parasitoids	0.295	0.081	0.223	0.190
		Phytophagous insects	0.022	0.898	0.079	0.647
CM	VO	Syrphidae	0.107	0.536	−0.141	0.413
	BTS	Predators	0.031	0.857	−0.156	0.363
		Parasitoids	0.093	0.588	−0.101	0.557
		Phytophagous insects	0.154	0.369	−0.026	0.881
WAA	WAA colonies	% shoots infested with WAA colonies	0.227	0.183	0.597	**<0.001**
		Predators	−	−	0.253	0.136
		Parasitoids	0.253	0.136	−	−

* Test statistics (Spearman correlation coefficients (ρ) and likelihood ratio (p)) are shown. Significant *p* values (*p* <0.05) are shown in bold.

## Supplementary Materials

The following are available online at https://www.mdpi.com/2075-4450/10/5/148/s1, Table S1: apple orchards surveyed in 2015 and 2016: locality, coordinates (UTM), year of planting and apple cultivars, Table S2: blooming period and percentage of plant coverage of the species present in the flower margin in 2015, Table S3: blooming period and percentage of plant coverage of the species present in the control margin in 2015, Table S4: level of identification, location, developmental stage of the arthropods found in the samplings and type of statistical analysis, Table S5: tests statistics (F-value (F)) and p (likelihood ratio) for the interaction between treatments (Year*Treatment) and Year for the number of: adult hoverflies, predators, parasitoids and phytophagous insects.
